# A Wearable Ground Reaction Force Sensor System and Its Application to the Measurement of Extrinsic Gait Variability

**DOI:** 10.3390/s101110240

**Published:** 2010-11-16

**Authors:** Tao Liu, Yoshio Inoue, Kyoko Shibata

**Affiliations:** Department of Intelligent Mechanical Systems Engineering, Kochi University of Technology, 185 Miyanokuchi, Tosayamada-Cho, Kami-City, Kochi 782-8502, Japan; E-Mails: inoue.yoshio@kochi-tech.ac.jp (Y.I.); shibata.kyoko@kochi-tech.ac.jp (K.S.)

**Keywords:** centre of pressure, gait variability, ground reaction force, obstacle avoidance, wearable force sensor

## Abstract

Wearable sensors for gait analysis are attracting wide interest. In this paper, a wearable ground reaction force (GRF) sensor system and its application to measure extrinsic gait variability are presented. To validate the GRF and centre of pressure (CoP) measurements of the sensor system and examine the effectiveness of the proposed method for gait analysis, we conducted an experimental study on seven volunteer subjects. Based on the assessment of the influence of the sensor system on natural gait, we found that no significant differences were found for almost all measured gait parameters (p-values < 0.05). As for measurement accuracy, the root mean square (RMS) differences for the two transverse components and the vertical component of the GRF were 7.2% ± 0.8% and 9.0% ± 1% of the maximum of each transverse component and 1.5% ± 0.9% of the maximum vertical component of GRF, respectively. The RMS distance between both CoP measurements was 1.4% ± 0.2% of the length of the shoe. The area of CoP distribution on the foot-plate and the average coefficient of variation of the triaxial GRF, are the introduced parameters for analysing extrinsic gait variability. Based on a statistical analysis of the results of the tests with subjects wearing the sensor system, we found that the proposed parameters changed according to walking speed and turning (p-values < 0.05).

## 1. Introduction

The quantitative analysis of gait variability using kinematics and kinetic characterisations can be helpful to medical doctors in monitoring patients’ recovery status in clinical applications. Moreover, these quantitative results may help to strengthen their confidence in the rehabilitation. Walking speed, stride length, the centre of mass (CoM) and the centre of pressure (CoP) have been considered as factors in the evaluation of walking gait [1–3]. According to one study on slip type falls [4], friction force was used to draw up important safety criteria for detecting safe gait, so the transverse components of ground reaction force (GRF) may provide important information for quantifying gait variability.

Many kinds of stationary systems such as force plates and instrumented treadmill devices are available to measure CoP and triaxial GRF [5–12]. Because a stationary force plate cannot measure more than one stride, in studies of continuous walking, a complex system consisting of many force plates and a data fusion method must be constructed [5–8]. Therefore, the force plate technology probably imposes some constraints on our ability to measure human movement and is not feasible for measurements in everyday situations. An instrumented treadmill or dynamometric platform formed by laying two force plates under a treadmill can overcome some limitations of the system with distributed multiple force plates in successive measurements of the GRF for gait evaluation [9,10]. However, a guide used to constrain the direction of the foot is necessary to ensure that subjects walk along a straight line, because if a human body segment motion analysis system is not available for a simultaneous measurement of the foot orientation, any technique based on force plates conventionally requires subjects to walk along a pre-defined specific path. Although gait variability can be assessed in straight walking [11], gait analysis concentrating solely on straight-line walking or running may not adequately interpret gait variability, because turning or walking direction changes probably have effects on extrinsic gait variability [12].

To overcome such limitations of stationary devices in GRF measurement, many researchers are developing wearable sensors attached to shoes [13–17]. Pressure sensors have been widely used to measure gaits and the distributed vertical component of GRF and to analyze the loading pattern on the plantar soft tissue during the stance phase of gait [13], but in these systems the transverse components of GRF (friction forces) which are one of the main factors leading to falling, have been neglected. By fixing two externally mounted sensors beneath the front and rear boards of a special shoe, researchers have developed an instrumented shoe for ambulatory measurements of CoP and triaxial GRF in successive walking trials [15], and the application of the instrumented shoe to estimate joint moments and powers of the ankle was introduced in [17]. The mounted sensor itself, having a height of 15.7 mm, increases the height and weight of the shoe, and affects normal walking gait. Moreover, its application study was restricted to human kinetics analysis using the spatio–temporal measurements of GRF and CoP.

In this paper, we describe a new wearable GRF sensor system which has a thin and light sole. Verification of the system’s measurements and evaluation of effect of the system to natural gait are presented. Moreover, we applied the multi-step data of CoP and triaxial GRF obtained from the sensor system to analyze extrinsic gait variability.

## 2. Methods

### 2.1. Wearable GRF Sensor System

**Figure 1.**
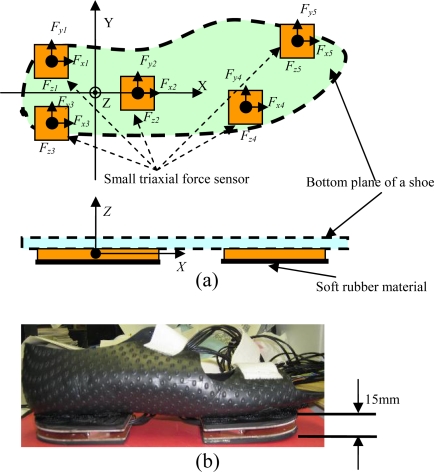
A wearable GRF sensor system constructed using five small triaxial force sensors. **(a)** Definition of sensors’ ordinate system and sensor mechanism; **(b)** Prototype of an instrumented shoe for right foot.

As shown in Figure 1, a wearable GRF sensor system was constructed using five small triaxial force sensors (USL06-H5-500N-C, weight: 15 g, size: 20 mm × 20 mm × 5 mm) made by TEC GIHAN CO., Japan. The GRF and CoP measured using the wearable sensor system were expressed in a global coordinate system which was located on the interface between the instrumented shoe and the ground. The origin of the global coordinate system was fixed to a point around the anatomical centre of the ankle when the sensor system was worn on the foot. The *x*-axis was chosen to represent the anterior-posterior direction on the interface plane contacting with the floor, which was based on landmarks from the shoe. The *z*-axis was made vertical, and the *y*-axis was chosen such that the resulting global coordinate system would be right-handed. By mounting the five triaxial sensors on an aluminum plate beneath the shoe, we can accurately align all five local coordinate systems defined for each triaxial sensor with the global coordinates. *F_xi_, F_yi_* and *F_zi_* (*I* = 1, 2, 3, 4 and 5) indicate triaxial forces measured by the distributed five triaxial sensors, and (*x_i_, y_i_*) is defined as the position of each triaxial sensor, for example, (*x_5_, y_5_*) indicates the position of the sensor placed on the forefoot [see Figure 1(a)]. The total weight of the sensor shoe is about 300 g, and the shoe size is 250 mm.

In this study, the mid-stance phase (foot-flat) during which the heel and forefoot are in contact with the ground, was evaluated to analyze extrinsic gait variability. All three components of GRF and CoP coordinates (*x_o_, y_o_, z_o_*) in the foot-flat phase could be easily calculated by using the triaxial forces measured using the five triaxial sensors. The calculation method is similar to that of our previously proposed measurement system [18].

A multi-channel data-logger was specially designed using a micro-computer (PIC 16F877A) system for the sensor system, and the data from the sensor system could be saved in an SRAM at a sampling rate of 100 Hz. The proposed algorithm for evaluating extrinsic gait variability was made using MATLAB software (The Mathworks, Natick, MA, USA). The off-line data analysis was performed by uploading data saved in the SRAM to a personal computer through a RS232 communication module. The small triaxial force sensors integrated in the shoe and the portable data-logger based on a micro-computer system were all low energy consumption devices, so the wearable sensor system could be powered using a 300 mAh (NiMH 30R7H) battery.

### 2.2. Experimental Methods

A combination system composed of a force plate EFP-S-2KNSA12 (KYOWA, Japan) and an optical motion analysis system Hi-DCam (NAC Image Tech., Japan) was used as a reference measurement system to validate the results of the developed sensor system. A low-pass filter with a cut-off frequency of 10 Hz was applied to the measurements of the reference system. By measuring orientations of the global coordinate system fixed on the developed sensor system using the optical motion analysis system, we transformed the stationary force plate measurements in their coordinate system to the global coordinate system of the sensor system, and compared the measurements of the developed sensor system with the reference system using a statistical method. Seven young volunteers (four men and three women: age = 28.5 ± 3.5 years, height = 168.5 ± 5.5 cm, weight = 63.4 ± 9.3 kg) were required to wear the sensor system to walk on the force plate, and the signals from the sensor system and the reference system were simultaneously sampled at 100 samples/s.

**Figure 2.**
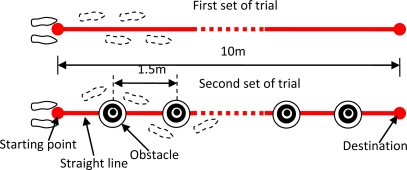
Two sets of trials designed in the experimental protocol. The distance between starting point and destination is 10 m for the two trial paths.

In order to apply the developed sensor system to extrinsic gait variability analysis, we designed a test experiment for the application of the sensor system by considering the effects of walking speed and turning on walking gait [12,21]. Subjects were asked to walk at three speeds including slow speed, preferred speed, and fast speed. The experimental protocol required two sets of trials for each subject who repeatedly walked for three times at each speed. As shown in Figure 2, in the first set of trials conducted to examine the effect of speed on extrinsic gait variability, a visible straight line was used to ensure straight walking in the trials for testing different walking speeds. In the second set of trials designed to analyze the effect of turning on extrinsic gait variability, subjects were required to turn around some obstacles when walking along the straight line to the same destination as in the first set of trials. The experiment locale selected was a 10 m long passageway, and each subject performed six walking trials designed in the experimental protocol. Each trial consisted of seven or eight strides.

### 2.3. Calculation Method

Gait evaluation based on analysis of temporal features of GRF and CoP has commonly been discussed on the basis of measurements of force plates fixed on the ground [19,20], in which limited steps and restricted walking conditions including limited speed and stride length make it difficult to implement statistical analysis with substantial gait data. Some wearable sensor systems are being developed to measure human motion and force without limitations on step and walking conditions, and a new evaluation method to analyze walking gait based on spatial parameters is introduced in this paper. GRF’s time sample matrix *G(t)* consists of a force vector *F(t)* composed of three components (*F_x_(t)*, *F_y_(t)*, *F_z_(t)*), and CoP’s coordinate function of *P(t)*, which gives two coordinates *x* and *y*, referring to the global coordinate system of the sensor system:

(1) $$G(t)=[F_{x}(t),F_{y}(t),F_{z}(t),P_{x}(t),P_{y}(t)]$$

where *t* ⊂ [*t_on_*,*t*_off_], and on/off subscripts mark the beginning/end of the foot-flat phase of gait.

To analyze all the successive foot-flat phases *G(t)* in a walking trial, two necessary conditions for detecting periods of heel and forefoot contact were applied to the vertical force component *(F_z1_(t)* + *F_z3_(t))* obtained from the two triaxial sensors on the heel and *F_z5_(t)* obtained from the triaxial sensor on the forefoot.

The first condition is to find the periods when *(F_z1_(t)* + *F_z3_(t))* and *F_z5_(t)* are non zero, so a binary mask was defined such that it has value ‘1’ during foot-flat periods, and ‘0’ otherwise. The mask (*M(t)*) can be calculated by defining the threshold (*C* = 50N)) of the magnitude signals of *(F_z1_(t)* + *F_z3_(t))* and *F_z5_(t)*:

(2) $$M(t)=\{\begin{array}{c} 1,\mathrm{if}\{(F_{z1}(t)+(F_{z3}(t))>C\&(F_{z5}(t))>C\}\\ 0,\mathrm{otherwise} \end{array}$$

Based on the two selection conditions above, the time sample matrix *G(t)* of every foot-flat phase in a successive walking trial could be defined as *G^k^(t)* in the period *t* ⊂ [*t^k^_on_*,*t^k^_off_* ] in which the resulting mask (*M(t)*) has a value equal to 1, and the superscript *k* indicates the number of steps. These time sample matrices were used to analyze walking gait:

(3) $$G^{k}(t)=\left[F_{x}^{k}(t),F_{y}^{k}(t),F_{z}^{k}(t),P_{x}^{k}(t),P_{y}^{k}(t)\right]$$

The general scheme of the evaluation method consists of two steps:

- CoP envelope curves calculated from CoP trajectories of each step during successive walking (if all the CoP trajectories are obtained in a successive trial);
- Measures of dispersion of triaxial GRF in the boundary area of the CoP envelope curves.

Envelope curves were used to simplify the gait evaluation algorithm by giving boundary conditions for integration calculation. In the first step (the calculation of the CoP envelope curves), two envelope curves including medial boundary function *EC^Medial^: (x^M^, y^M^)* and lateral boundary function *EC^Lateral^: (x^L^, y^L^)* were determined using CoP data in *G^k^(t)*, while *x_posterior_* and *x_anterior_* were defined as the limitation values of *x* in the two functions (*x^M^* ⊂ [*x_posterior_*, *x_anterior_*] and *x^L^* ⊂ [*x_posterior_*, *x_anterior_*]). Because the 2D coordinates of CoP were captured in a spatio-temporal form, we must reconstruct CoP trajectories for each step using a spline function to estimate all the CoP functions referred to *x*-axial coordinates. The functions of two envelope curves referring to the defined global coordinate system could be obtained by extracting boundary values for all the CoP functions (referring to Figure 5 in results section).

The second step is based on the results obtained from CoP envelope curves estimated in the first step. We adopted the area (*A_cop_*) of the CoP distribution across the foot-plate as one of the parameters used for evaluating extrinsic gait variability. Moreover, we calculated the statistical data of the triaxial GRF and its correlation with the CoP distribution in the global coordinate system. To examine extrinsic gait variability in the three directions, we calculated the variability of the *x-*, *y-* and *z-*axial GRF. Variability data for each directional component was expressed as the average coefficient of variation (*ACV*) across all step data collected within each trial, and were calculated using the following equations:

(4) $$A_{\mathrm{Cop}}=\int_{x_{\mathrm{posterior}}}^{x_{\mathrm{anterior}}}\int_{y^{M}}^{y^{L}}\mathrm{dxdy}$$

(5) ACV=∫xposteriorxanterior∫yMyLS.D.(F)F¯dxdyAcop

where *S.D.(F)* and *F̄* are the standard deviation and the mean force distribution in the area between the medial boundary *EC^M^ (x^M^, y^M^)* and the lateral boundary *EC^L^* *(x^L^, y^L^)* of CoP respectively. In this research, we calculated ACV^X,Y,Z^ : [ACV^X^, ACV^Y^, ACV^Z^ ] for three-directional GRF to evaluate walking extrinsic gait variability.

### 2.4. Analysis Methods

Before validating the accuracy of the sensor system, we used the parameters including stride length (SL), stride width (SW), maximum lateral foot excursion (ME), single stance time (SST), double stance time (DST), stride time (ST), maximum GRF (MaxF) and minimum GRF (MinF) as proposed by Liedtke [16] to assess the influence of the sensor system on natural gait. When the seven subjects walked across the force plate with normal shoes and walked with the sensor system respectively, we measured foot motion and triaxial GRF ten times using the reference system. Each parameter was determined for a stride and averaged over ten trials with normal shoes, and then compared with the wearable GRF system in place under the same conditions.

Statistical analyses were performed to determine the effects of walking direction and speed on extrinsic gait variability, which are presented as mean ± standard deviation (SD). Linear mixed effects models [22] were used to assess whether differences in the proposed parameters of *A_cop_*, ACV^X^, ACV^Y^ and ACV^Z^ by walking condition (speed and direction) were statistically significant (p < 0.05). The fixed effects for this study were the average differences in *A_cop_*, ACV^X^, ACV^Y^ and ACV^Z^ for each walking condition using a repeated measures analysis of variance (ANOVA).

## 3. Experimental Results

### 3.1. Validation of the Wearable Sensor System

**Figure 3.**
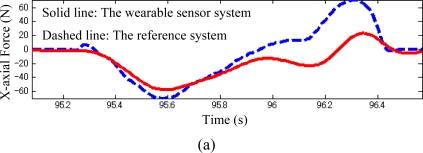

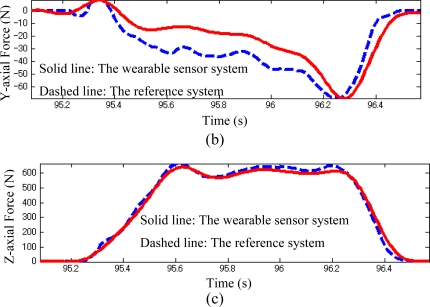
Triaxial GRF measured by the wearable sensor system (solid line) and referenced system (dashed line) during walking trial. **(a)** Comparison results of x-axial force (anterior-posterior direction); **(b)** Comparison results of *y*-axial force (medio-lateral direction); **(c)** Comparison results of *z*-axial force (vertical direction).

**Figure 4.**
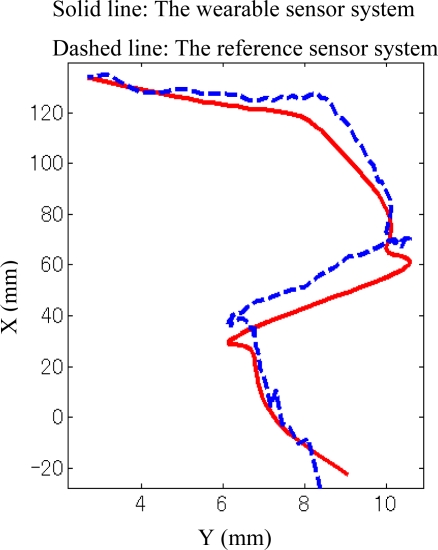
Centre of pressure (CoP) measured by the wearable sensor system (solid line) and reference sensor system (dashed line) referred to a global coordinate system. and referenced sensor system (dashed line) during walking trial.

As shown in Figures 3(a–c), the comparisons of the three components of GRF measured by the developed sensor systems and the reference sensor system were demonstrated in a representative walking trial. The results show a good correspondence between the newly developed sensor and the reference devices, which was examined by root mean square (RMS) difference for the seven subjects’ trials. The average of the RMS difference and standard deviation of the two transverse components (*x-* and *y-* axis) and the vertical component (*z-*axis) of the GRF were 5.0 ± 0.7 N, 10.0 ± 0.3 N, and 7.0 ± 0.4 N respectively, corresponding to 7.2% ± 0.8% and 9.0% ± 1% of the maximum of each transverse component and to 1.5% ± 0.9% of the maximum vertical component of GRF. The verification results of the CoP trajectories are given in Figure 4.

The RMS distance between both CoP measurements was 2.1 ± 0.4 mm, corresponding to 1.4% ± 0.2% of the length of the shoe. In order to examine whether these reported differences are statistically significant, we quantified the differences between the two measurement systems using the ASTM standard which reports differences in terms of bias and precision. The terms repeatability limit and reproducibility limit are used as specified in Practice E 177 of the ASTM standard [23].

The 95% repeatability limit (within subject) and 95% reproducibility limit (between subjects) of the triaxial GRF measurements are 2.5 N and 2.5 N for *x-*axial GRF; 1.3 N and 2.0 N for *y-*axial GRF; and 1.7 N and 1.7 N for *z-*axial GRF. The 95% repeatability limit (within subject) and 95% reproducibility limit (between subjects) of the CoP measurement are 1.5 mm and 2.0 mm. The respective standard deviations of repeatability standard deviation and reproducibility standard deviation (in Practice E 691 of the ASTM standard [24]) among test results may be obtained by dividing the above limit values by 2.8.

### 3.2. Effect of the Sensor System on Natural Gait

The parameters used to assess the effect of the sensor system on gait were averaged over ten trials per subject. An overview is presented in Table 1, and none of the mean and SD of the parameters for the two shoe types showed significant differences (p-values < 0.05). Stride length (SL), stride width (SW), maximum lateral foot excursion (ME), single stance time (SST), double stance time (DST), stride time (ST), maximum GRF (MaxF) and minimum GRF (MinF) averaged over all subjects differed by 13.6 mm, 6.4 mm, 1.8 mm, 0.05 s, 0.02 s, 0.09 s, 11.4 N and 7.2 N between normal shoes and the sensor system respectively. The repeatability standard deviation and reproducibility SD of the differences between the two shoe types for the eight evaluation parameters are 0.5 mm and 0.8 mm for SL; 0.7 mm and 0.7 mm for SW; 0.3 mm and 0.5 mm for ME; 0.02 s and 0.03 s for SST; 0.03 s and 0.03 s for DST; 0.05 s and 0.05 s for ST; 1.1 N and 1.7 N for MaxF; and 0.8 N and 1.0 N for MinF.

### 3.3. Extrinsic Gait Variability

**Figure 5.**
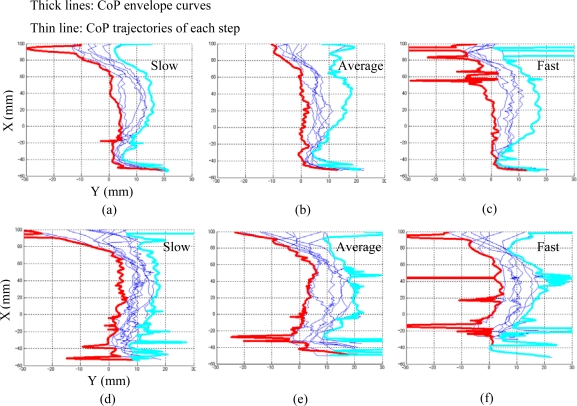
Representative plots of CoP envelope curves (thick lines) and CoP trajectories for each step in the successive walking trials. CoP trajectories of the six trials were given in the six sub-plots in which the horizontal axis and vertical axis were defined as medial-lateral direction and anterior-posterior direction respectively. Moreover, the left and right envelope curves were plotted using a widening solid line for each trial. **(a)**, **(b)** and **(c)** are the results of the first set of walking trials with a lead line under slow, preferred and fast speed respectively. **(d)**, **(e)** and **(f)** are the results of the second set of walking trials with obstacles.

Using the developed algorithm, we extracted GRF data for foot-flat gait phase of each step from the measured spatio-temporal gait data. The CoP envelope curves were calculated from the CoP trajectories of the extracted intervals. The size of the spreading area of the trajectories of CoP on the foot-plate of different steps for the walking with obstacles was greater than that for the walking without obstacles, at all walking speeds. Moreover, the area between the calculated CoP envelope curves increased with accelerating movements (see Figure 5). A number of spikes due to the anticipated noise interference nearing the beginning and end of each step appeared on the calculated envelope curves, but these never affected the accuracy of the variability algorithm, because the integration calculations in the area of the envelope curves were not sensitive to these less accurate points.

**Figure 6.**
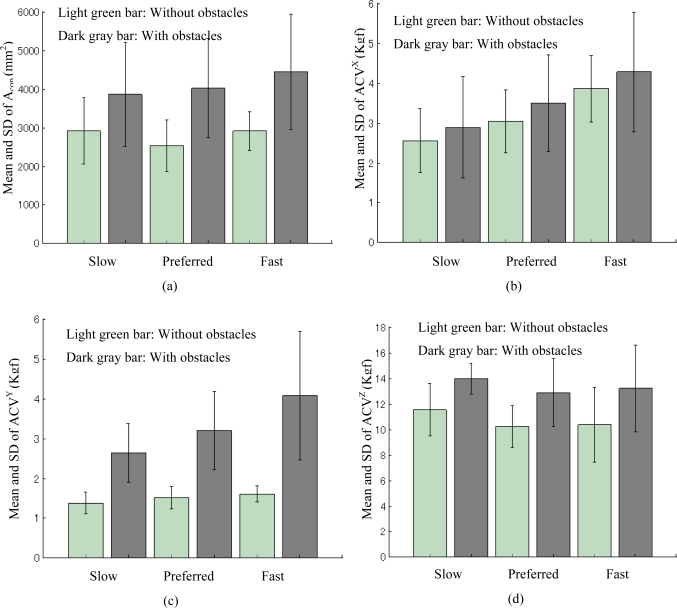
Statistical comparison of the evaluation parameters’ (Acop, ACV^X^, ACV^Y^ and ACV^Z^) mean and standard deviations (SD) for all the subjects.

**Figure 7.**
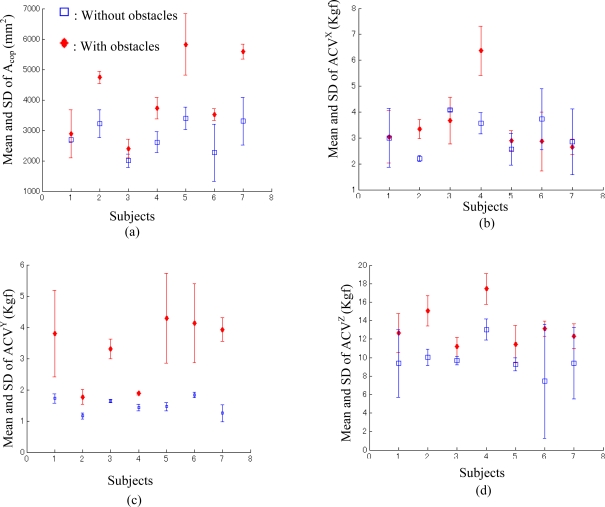
Statistical comparison of evaluation parameters’ (Acop, ACVX, ACVY and ACVZ) mean and standard deviations (SD) of each subject for the three selected speeds required in the two sets of trials. The square markers and deviation scopes represent the evaluation parameters’ mean and standard deviations (SD) in the first set of trials where subjects were required to walk along a lead line; and the diamond markers and deviation scopes are used to represent the second set of trials where subjects were asked to walk bypassing some obstacles.

The mean and SD of Acop and ACV for walking without obstacles compared to walking with obstacles at three walking speeds (slow, average and fast) for the seven subjects in the three repeated trials are shown in Table 2. The means of Acop and ACV of walking with obstacles were greater than the means of Acop and ACV of walking without obstacles for all three speeds (slow, average and fast). Moreover, slow speed was significantly less than average speed (0.6 ± 0.2 m/s, 1.0 ± 0.1 m/s, respectively; p < 0.0009), and average speed was significantly less than fast speed (1.7 ± 0.1 m/s, p < 0.0006). We also found that the means of ACV of *x-* and *y-* directions (the two transverse directions) became greater with increase in walking speed [see Figures 6 (b,c)], but a similar trend was not found for the means of Acop and ACV of *z-*direction (vertical direction) [see Figure 6 (a,d)]. As shown in Figure 7, to examine the effect of turning on extrinsic gait variability for individual subjects we compared the mean and SD calculated by the parameters of Acop and ACV of the three speeds under each condition (walking without obstacles and walking with obstacles). The means of Acop and ACV of walking with obstacles were greater than the means of Acop and ACV of walking without obstacles for each subject.

## 4. Discussion

The force measurements by the sensor system in the *x-* and *y-* axes demonstrated both amplitude and phase shifts from the reference measurements (Figure 3), and the discrepancy in the CoP trajectory was slightly smaller than that described in the results reported by Veltink [15]. The output signals of the force plate and motion capture data in the reference sensor system were filtered by a low-pass filter with a cut-off frequency of 10 Hz, which imposes phase shifts in the measurements between the two systems. The most likely source of amplitude error in the triaxial GRF measurement was from the orientation estimate of the sensor system using the optical motion analysis system Hi-DCam, which could only implement 3D position measurements with about 1 mm accuracy. As another reason, five triaxial force sensors mounted on an aluminum plate were used in the design of the prototype for the wearable sensor system to measure CoP and triaxial GRF. If we mount more triaxial force sensors beneath the shoe, the precision of the new sensor system will be improved even further, because a smaller torque is produced on each triaxial force sensor. Moreover, the large stiffness of the plate could obviously influence the mechanical characteristics of the shoe, if we increase the number of the sensors distributed under the plate, the thickness of the plate can be optimized to make the sensor system have the same bending stiffness as the sole.

Significant differences between instrumented and normal shoes have been found in the maximum ground reaction force [16], in which the maximum ground reaction force averaged over all subjects has been show to differ by up to 56 N between instrumented and normal shoes. However, as has been shown in Table 1, there were not significant differences between our sensor system and a normal shoe (p-values < 0.05), probably because we adopted small triaxial force sensors (size: 20 mm × 20 mm × 5 mm) in the sensor system design, which allows natural or near-natural gait.

As a research application, some sensor systems have been used to evaluate the effects of walking speed and walking direction change on extrinsic gait variability. Kinematic gait parameters calculated using joint motion trajectory have been widely adopted to analyze gait variability [21,25,26], but an optical motion capture system limited to laboratory use has usually been used to measure joint motion. To overcome the disadvantage of the stationary motion analysis system, some wearable sensor systems based on inertial sensors have been developed to assess spatio-temporal parameters during unconstrained walking [27–29]. However, in this paper we propose a new method to implement extrinsic gait variability assessment using CoP and triaxial GRF in a non-laboratory environment. This method may be a supplement to the existing method based on kinematic gait parameters.

The effects of walking speed and turning upon gait must be considered when interpreting the effectiveness of these new definitions for extrinsic gait variability. Turning or walking direction changes affect lower limb kinematics, kinetics, and step length [30], which may influence gait variability. As shown in Figure 7, the means of Acop and ACV markedly increase for all the subjects in the trials of walking around obstacles when compared with those trials where subjects were asked to walk along a straight line. Moreover, to examine the effectiveness of the parameters in evaluating turning effects for individual subjects, we compared the mean and SD of Acop and ACV of the three speed conditions between the walking condition without obstacles and the condition with obstacles (Figure 7). Smaller means of Acop and ACV indicate a lower variability of walking dynamics, which can be used to evaluate the effects of turning on extrinsic gait variability.

Walking speed affects kinematics, double-support time, step width and other clinical correlates of stable walking [31], because faster walking speed increases the segmental momentum thereby requiring greater effort to attenuate kinematic disturbances. As shown in Figures 6(b,c), we found that ACV^X^ and ACV^Y^ augment when subjects increase walking speed, so the average coefficients of variation (ACV) of the *x-*axis force (anterior–posterior friction force) and the *y-*axis force (medial–lateral friction force) can be applied to evaluate extrinsic gait variability induced by different walking speeds, because the transverse components of GRF or friction forces connected with body segment motion are sensitive to human walking speed. Figures 6(a) and (d) show that the parameters Acop and ACV^Z^ obtained from the vertical component of GRF could not clearly reflect the walking speed changes, so they cannot be used to assess speed effects on extrinsic gait variability during level walking.

## 5. Conclusions

A wearable GRF sensor system to measure CoP and triaxial GRF in a number of walking trials was developed. Natural gait was almost never affected by the sensor system in the ambulatory GRF measurements. The 95% repeatability limit (within subject) and 95% reproducibility limit (between subjects) of the triaxial GRF measurements is 2.5 N and 2.5 N for *x-*axial GRF; 1.3 N and 2.0 N for *y-*axial GRF; and 1.7 N and 1.7 N for *z-*axial GRF. The 95% repeatability limit (within subject) and 95% reproducibility limit (between subjects) of the CoP measurement is 1.5 mm and 2.0 mm. A new method based on measurements from the sensor system gave a group of new evaluation parameters for quantifying extrinsic gait variability. The statistical analysis of GRF data extracted from successive gait trials may be more suited to the evaluation of gait for ambulatory or wearable force systems that can continuously measure human force and motion without restriction to a laboratory environment. In order to verify the effectiveness of the evaluation parameters of Acop, ACV^X^, ACV^Y^ and ACV^Z^ in walking gait analysis, we implemented an experimental study of a group of healthy subjects who were required to walk under the designed experimental protocol. According to our statistical analysis, the proposed parameters Acop, ACV^X^, and ACV^Y^ increased with the increase of walking speed (p-values < 0.05), but no similar trend was found for ACV^Z^ with the speed changes. All the parameters of walking with obstacles were greater than walking without obstacles for all three speeds, and these comparisons were statistically significant (see Table 2). The hypothesis that extrinsic gait variability can be quantified by calculating the variability of the CoP and GRF measured using the sensor system in a multi-step walking trial was therefore supported.

Although good results were obtained for validation and extrinsic gait variability analysis, the sample size for the application was limited (seven healthy subjects), and the variability was assessed on the aspect of obstacle-avoidance. In future research, experiments on more diverse subjects and various walking conditions will be necessary to support clinical applications of the sensor system.

## Figures and Tables

**Table 1.** The mean and standard deviation of the gait parameters including stride length (SL), stride width (SW), maximum lateral foot excursion (ME), single stance time (SST), double stance time (DST), stride time (ST), maximum GRF (MaxF) and minimum GRF (MinF) for all subjects (p-values < 0.05).

		**SL (mm)**	**SW (mm)**	**ME (mm)**	**SST (s)**	**DST (s)**	**ST (s)**	**MaxF (N)**	**MinF (N)**

**Normal shoes**	**Means ± SD**	1,440.9 ± 2.0	83.8 ± 1.7	25.3 ± 1.1	0.83 ± 0.08	0.16 ± 0.04	1.20 ± 0.09	709.5 ± 1.3	540.9 ± 1.9
**The sensor system**	**Means ± SD**	1,427.3 ± 1.3	90.2 ± 1.0	27.1 ± 0.5	0.88 ± 0.03	0.18 ± 0.06	1.11 ± 0.05	689.1 ± 2.2	533.7 ± 1.2

**Table 2.** The four parameters (mean ± SD) for the three walking speeds (slow, preferred and fast) at two sets of walking trials: without obtacles and with obstacles with corresponding p-values. Calculated evaluation parameters including CoP distribution area (*A_cop_*) in mm^2^ and the average coefficient of variation (*ACV*) of three directional forces in the two sets of walking trials implemented on seven subjects. *ACV^X^, ACV^Y^* and *ACV^Z^* are three directional components of *ACV*. Statistical significance was set at p < 0.05.

		Without obtacles	With obstacles	p-value
*A_cop_* × *10^3^*	Slow	2.9 ± 0.9	3.9 ± 1.3	0.001
	Preferred	2.5 ± 0.7	4.0 ± 1.3	0.007
	Fast	2.9 ± 0.5	4.4 ± 1.5	0.021
*ACV^X^*	Slow	2.6 ± 0.8			2.9 ± 1.3	0.013
	Preferred	3.0 ± 0.8			3.5 ± 1.2	0.009
	Fast	3.9 ± 0.8			4.3 ± 1.5	0.018
*ACV^Y^*	Slow	1.4 ± 0.3			2.6 ± 0.7	0.011
	Preferred	1.5 ± 0.3			3.2 ± 1.0	0.019
	Fast	1.6 ± 0.2			4.1 ± 1.6	0.023
*ACV^Z^*	Slow	11.5 ± 2.1			13.9 ± 1.2	0.032
	Preferred	10.2 ± 1.6			12.8 ± 2.6	0.045
	Fast	10.3 ± 2.9			13.2 ± 3.4	0.034
